# Metabolic risk management, physical exercise and lifestyle counselling in low-active adults: controlled randomized trial (BELLUGAT)

**DOI:** 10.1186/s12889-017-4144-8

**Published:** 2017-03-14

**Authors:** Assumpta Ensenyat, Gemma Espigares-Tribo, Leonardo Machado, Francisco José Verdejo, Rosa Rodriguez-Arregui, José Serrano, Marta Miret, Gisela Galindo, Alfonso Blanco, Josep-Ramon Marsal, Susana Sarriegui, Xenia Sinfreu-Bergues, Noemi Serra-Paya

**Affiliations:** Institut Nacional d’Educacio Fisica de Catalunya - Campus Lleida, Lleida, Catalonia Spain

## Abstract

**Background:**

The primary aim of this study is to evaluate the effectiveness of different doses (intensity) of supervised exercise training — concomitant with lifestyle counselling — as a primary care intervention tool for the management of metabolic syndrome risk factors in low-active adults with one or more such factors (programme name in Catalan: Bellugat de CAP a peus).

**Methods/Design:**

Three-arm, randomized controlled clinical trial implemented in the primary care setting, with a duration of 40 weeks (16 weeks intervention and 24-week follow-up).

Adults aged 30 to 55 years with metabolic risk factors will be randomized into three intervention groups: 1) aerobic interval training (16 supervised training lessons) plus a healthy lifestyle counselling programme (6 group and 3 individual meetings); 2) low-to-moderate intensity continuous training (16 supervised training lessons) plus the same counselling programme; or 3) the counselling- programme without any supervised physical exercise.

The main output variables assessed will be risk factors for metabolic syndrome (waist circumference, blood pressure, and levels of plasma triglycerides, high-density lipoproteins and glucose), systemic inflammation, cardiorespiratory fitness, physical activity and sedentary behaviour, dietary habits, health-related quality of life, self-efficacy and empowerment. Economic factors will also be analysed in order to determine the cost-effectiveness of the programme. These variables will be assessed three times during the study: at baseline, at the end of the intervention, and at follow-up. We estimate to recruit 35 participants per group.

**Discussion:**

The results of this study will provide insight into the immediate and medium-term effects on metabolic risk and lifestyle of a combined approach involving aerobic interval training and a multidisciplinary behavioural intervention. If effective, the proposed intervention would provide both researchers and practitioners in this field with a platform on which to develop similar intervention programmes for tackling the repercussions of an unhealthy lifestyle.

**Trial registration:**

Clinical trials.gov. NTC02832453. Registered 6 July 2016 (retrospectively registered).

## Background

Metabolic syndrome consists of a cluster of risk factors for cardiovascular disease that comprises abdominal obesity, impaired glucose tolerance, hypertension and dyslipidemia. When these factors appear together the cardiovascular risk rises substantially. In Spain, the prevalence of metabolic syndrome is 42.1% in men and 32.3% in women, and it increases with age [1].

A considerable body of epidemiological evidence indicates that the most prevalent diseases in developed societies, including metabolic syndrome, are closely linked to lifestyle factors, notably smoking, unhealthy dietary habits, physical inactivity and stress. The increasing prevalence of metabolic syndrome and its negative impact on individuals and society has been mainly attributed to a decline in regular physical activity, an increase in sedentary behaviour and changes in dietary habits. Given that these factors are all modifiable they may be reversed through interventions focussing on behavioural change. Changes in lifestyle can have a positively effect on the prevalence of cardiovascular risk factors and, therefore, on that of metabolic syndrome and cardiovascular diseases [2].

In an attempt to tackle the problem of unhealthy lifestyles, the World Health Organisation (WHO) developed a Global Strategy on Diet, Physical Activity and Health, the aim of which was to control and prevent non-communicable diseases or their risk factors [3]. Physical activity has a key role to play in any such strategy. However, despite investment in the promotion of physical activity, the degree to which people engage in regular weekly exercise remains worryingly low. According to the Special Eurobarometer 412 [4], 54% of European Union citizens interviewed had not, during the previous week, done any physical activity of vigorous intensity, and 44% had not done any exercise of moderate intensity. In addition to the lack of moderate-to-vigorous physical activity, about two-thirds (69%) of respondents also stated that they spent between 2.5 and 8.5 h sitting during waking hours, and 11% sat for more than 8.5 h.

The health benefits of regular physical exercise in terms of preventing and treating many non-communicable diseases are well-established [5]. They include positive effects on single metabolic risk factors, such as normalization of blood pressure and improvements in body composition, hyperlipidaemia and insulin resistance [5]. Physical exercise also increases cardiorespiratory and muscular fitness. Regarding metabolic syndrome as a whole, Pattyn and colleagues [6] recently conducted a meta-analysis and concluded that dynamic physical exercise has beneficial effects on the majority of metabolic risk factors (abdominal obesity, high-density lipoprotein cholesterol (HDLc), systolic and diastolic blood pressure), as well as on other factors such as body mass index (BMI) or maximal oxygen uptake (VO_2max_). However, they also highlight that the most favourable modality/dose of exercise (dynamic versus static; low-to-moderate intensity versus high intensity) has yet to be fully elucidated.

Present guidelines for physical exercise as a healthy behaviour are based around continuous training methods (30–60 min/session, low-to-moderate intensity, 2–7 sessions per week) [5, 7, 8]. The WHO [9] recommends that individuals aged 18–65 should accumulate a minimum of 150 min per week of moderate-intensity physical activity or, alternatively, 75 min per week of vigorous physical activity, or a combination of both.

However, although moderate-intensity exercise is sufficient to reduce cardiovascular risk, it has been suggested that higher intensity exercise offers greater benefits [10–12]. Accordingly, the health setting has recently witnessed a growing interest in interval training methods, particularly high-intensity interval training (HIIT). When the aim of this method is to stimulate preferentially the aerobic system over the anaerobic one, it is referred to as aerobic interval training [13]. Aerobic interval training (AIT) consists of repeated bouts, either short (<45 s) or long (1 to 8’), of fairly high-intensity exercise (equal or superior to the maximal lactate steady-state velocity) interspersed with recovery periods.

To date, several studies have analysed the effects of AIT in individuals with cardiovascular risk factors or with diseases such as metabolic syndrome, ischaemic cardiopathy or cardiac insufficiency. Results from these studies suggest that AIT produces a greater improvement in VO_2max_ than does traditional continuous training (TCT). The effect has been described in recreationally active individuals [14], as well as in patients with coronary artery disease [15–17] or metabolic syndrome [18, 19]. It has also been shown that AIT reduces the prevalence of metabolic risk factors [15, 17, 19, 20]. The positive effects of AIT on metabolic risk factors involve changes in body composition, ventricular and vascular functions, plasmatic adiponectin and glucose intolerance. Studies comparing the effects of different training methods generally report better results after AIT and suggest that this method optimizes the time necessary to induce muscular, metabolic and cardiorespiratory effects in the medium and long term [17, 19, 20].

Although these studies support the efficacy of AIT for reducing metabolic risk factors, its effectiveness has yet to be fully demonstrated. One of the difficulties faced when seeking to replicate in practice the evidence obtained in controlled studies is that individuals are less constrained and become more responsible for their own compliance with physical exercise. As Rankin [10] states, physical exercise will have no benefits unless the individual actually does it. Accordingly, there is evidence to suggest that the most effective interventions, among those aiming to promote healthier behaviour, are those that not only involve scheduled physical activity and exercise but which also offer lifestyle counselling, including advice on dietary habits and how to reduce daily sedentary time, along with tools that can empower individuals towards behaviour change.

Detractors of high intensity exercise claim that HIIT is not an appropriate or sustainable form of exercise for public heath guidelines because it is unlikely to induce changes in the population as a whole [21]. It is argued that HIIT will be associated with negative feelings and lack of enjoyment, leading therefore to poor compliance. Biddle, for example, claims that as a public health tool, higher intensity exercise may not be better if people are less willing to do it [21]. However, AIT is a form of HIIT in which exercise intensity is not maximal. This feature may explain why, contrary to what might be expected given the low physical condition of the participants in some studies, AIT sessions have been well tolerated by sedentary individuals with or without concomitant diseases [17, 19, 20, 22, 23]. Indeed, no adverse effects have been reported [10], even in patients with heart failure [12], because the intensity is tailored to the individual.

In addition, participants in AIT interventions report greater satisfaction than do people in TCT interventions. This could be attributable to the variable nature of the session, in comparison with the monotony of TCT sessions, as well as to the fact that the intensity of intervals promotes a greater sense of challenge. These aspects reflect the potential of AIT to improve adherence to exercise interventions [23]. Although in some individuals AIT may trigger a feeling of displeasure and, hence, impair their confidence and adherence [21], factors such as the optimization of time spent on training sessions and the associated satisfaction of participants may contribute to the consolidation and long-term persistence of an active lifestyle.

To our knowledge, although the efficacy of AIT in relation to cardiometabolic risk factors has been established in several studies, there are no data on the effectiveness of AIT when implemented in conjunction with lifestyle counselling as an intervention in the primary care setting.

The primary aim of this study is to evaluate the effectiveness of different *doses* (intensities) of *supervised* exercise training (AIT and TCT), both concomitant with lifestyle counselling, and to compare this with lifestyle counselling (COU) alone as a primary care intervention tool for the management of metabolic syndrome risk factors in low-active adults with one or more such factors.

Secondary aims of the study are to investigate the effects of these interventions on systemic inflammation and adipose tissue function, cardiorespiratory fitness, physical activity and sedentary behaviour, dietary habits, empowerment related to health, self-efficacy in relation to physical activity and dietary habits, as well as the cost-effectiveness of the intervention with regard to health-related quality of life.

The study will also provide insights into the extent to which any observed changes persist in the medium term.

The general hypothesis is that among adults with risk factors for metabolic syndrome, those who participate in interventions designed to promote a healthy lifestyle through a combination of lifestyle counselling and supervised physical exercise of vigorous intensity will present greater improvements in terms of metabolic risk, physical condition, physical activity/sedentary behaviours and psychological parameters at the end of the intervention and at 24-week follow-up than will participants in interventions that combine lifestyle counselling with physical exercise of low-to-moderate intensity or those based exclusively on lifestyle counselling.

## Methods

### Trial design

**Fig. 1:**
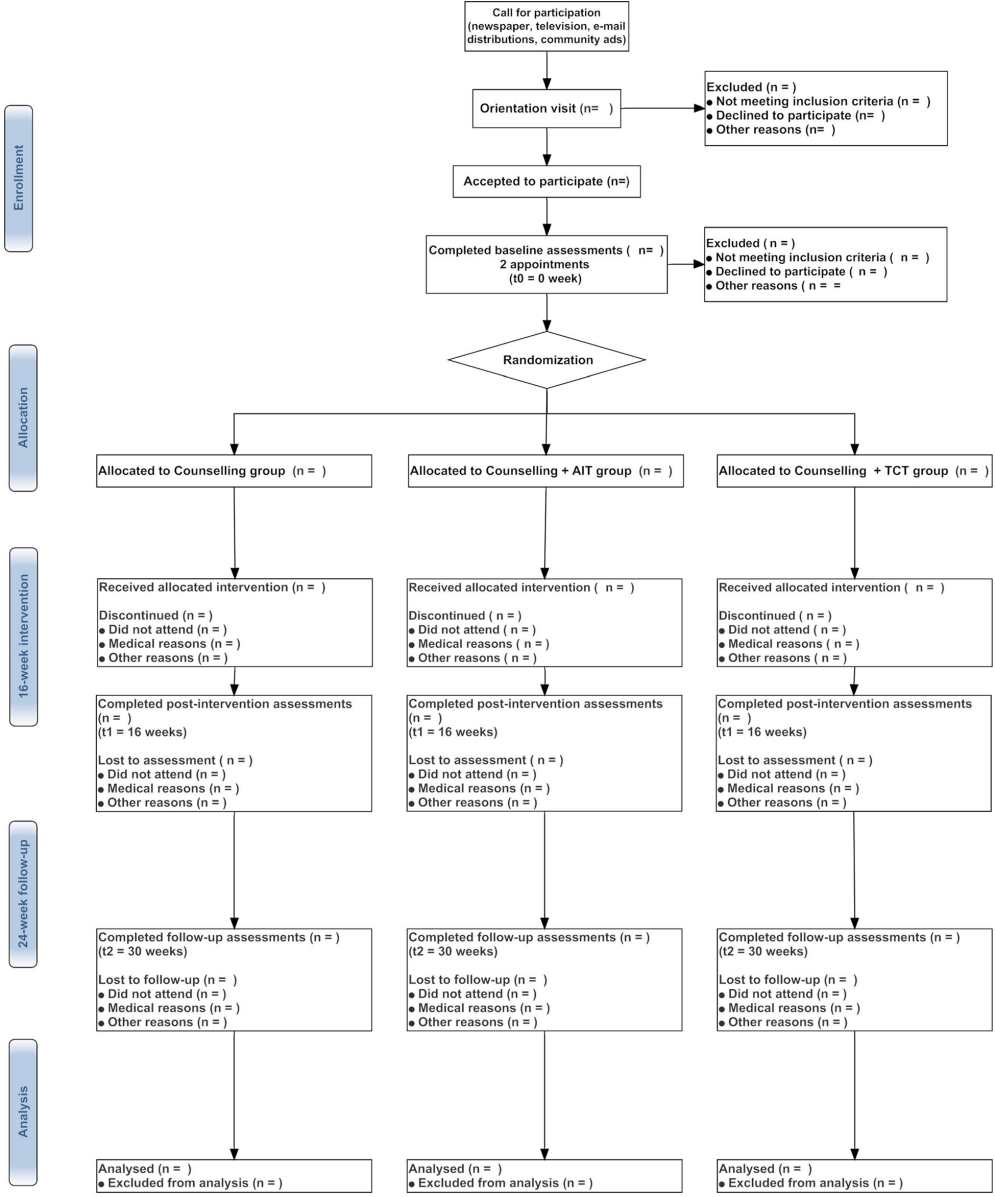
Bellugat flow chart

This is a three-arm *(dose–response exercise*) randomized controlled clinical trial implemented in the primary care setting over a period of 16 weeks and with 24-week follow-up (Fig. 1).

The study will include two supervised exercise groups with two different doses of exercise intensity (AIT and TCT), and a non-exercise control group (COU). All groups will be given lifestyle counselling.

### Participants

#### Eligibility criteria of participants

To be eligible, individuals should meet all the following inclusion criteria and none of the exclusion criteria.

##### Inclusion criteria

- 30 to 55 years-old.
- Having one or more risk factors for metabolic syndrome^1^ (waist circumference >94.5 cm for men and >89.5 cm for women; blood pressure ≥130/85 mmHg; triglycerides in plasma ≥150 mg/dL; HDLc in plasma <40 mg/dL for men and <50 mg/dL for women; fasting glycaemia ≥100 mg/dL).
- Low active (achieving less than 150 min/week of moderate-to-vigorous physical activity or to have not participated in any supervised exercise programmes for at least the last 24 weeks).
- Accept and sign written informed consent.
- Accept the randomized group assignment.

##### Exclusion criteria

- Morbid obesity (BMI ≥ 40).
- A past/current history and/or evidence through physical examination or laboratory findings of significant cardiovascular, respiratory, neuromuscular or psychiatric disease/disorder.
- Diseases/disorders that may contraindicate performing physical exercise or a stress test [7].

#### Enrolment, screening and allocation

Participants will be recruited through primary healthcare centres, including physician referrals and advertisements through the media, community centres and electronic mailing. Interested individuals will be invited to an information session, where an in-depth detailed explanation of the study requirements and expectations will be given. Any questions will be answered and doubts clarified. Potential eligibility will be checked/confirmed by interview, obtaining information about age, past and current physical activity, medical history and medication use. Eligible participants who wish to participate in the study will be asked to sign written informed consent.

After providing written informed consent individuals will be given an identification number. Participants will be allocated to one of the two supervised physical exercise groups (AIT or TCT) or to the control group (COU), using a computer generated randomized list which allocates at random the identification number of the participant to a treatment group. The allocation sequence will be generated by the IDIAP and will be conducted without influence of the principal investigators.

#### Blinding

Due to the nature of the intervention, blinding of participants will not be possible as each of them will be aware of the kind of physical exercise they are engaged in. However, during data analysis researchers will be blinded to the intervention allocated to each participant.

#### Sample size

At least 105 individuals will be recruited for this study. Based on previous studies [20], a sample of this size would provide a beta risk of 0.2 and an alpha risk of 0.05 when using a bilateral contrast to detect a mean change in waist circumference of 6.0 cm (SD 5.3). Assuming a 20% drop-out rate at follow-up, we aim to recruit 35 individuals for each intervention group.

#### Ethical implications

The study will be carried out in accordance with the principles of the Declaration of Helsinki, and subsequent revisions, as well as with the Guidelines for Good Practice in Primary Care Research of the Jordi Gol Primary Care Research Institute (IDIAP).

The research protocol has been reviewed and approved (P15/122) by the Institutional Board (CEIC) of the IDIAP, and written informed consent will be obtained from all participants prior to their inclusion in the study. Specifically, all individuals will give their written informed consent in accordance with the principles of the Declaration of Helsinki and the dispositions established in Spanish law (Chapter II, article 7, Royal Decree 223/2004 of 6 February 2004); the consent form will specify that participation in the study is voluntary and that participants may withdraw at any time. This information will be provided both orally and in writing.

In this study, only researchers and health care professionals will have access to participants’ data.

The study methods are consistent with the CONSORT guidelines for the reporting of randomized trials [24].

### Trial intervention

The total duration of the trial will be 40 weeks, comprising a 16-week intervention phase and follow-up 24 weeks later. During the intervention phase all groups (AIT, TCT and COU) will be offered lifestyle counselling, alongside which the two exercise groups (AIT and TCT) will take part in a supervised physical exercise programme.

#### Counselling programme

The counselling programme consists of six, 50-min group sessions and three individual sessions lasting 40–50 min each. All these sessions will be conducted at the primary healthcare centre by a nurse trained in physical activity, nutrition and psychological strategies for behaviour change. An interactive participants’ guide has been published with the aim of reinforcing the counselling programme [25]. As a general rule, participants will be required, before the sessions, to read certain information (reports or chapters from the guide) or to reflect on the process of behaviour change. These aspects will then be discussed during the group or the individual meetings. Table 1 shows a detailed outline of the aims and activities of these meetings.

**Table 1:** Aims and activities of healthy lifestyle counselling sessions for all interventions

N	Aims of the session	Example of meeting activities/actions *(Participant actions unless otherwise specified)*	
1^st^ Group Session (week 1)	To explain the programme To connect with the rest of the group and establish the dynamics of the meetings To become aware of the benefits of a healthy lifestyle To establish personal goals limiting the time spent in sedentary behaviours	Before the session	1. Browse the BELLUGA’T guide promoting a healthy lifestyle. 2. Review the schedule and the programme plan.
		During the session	Leader’s action: 1. Present the programme: structure, dates, goals, strategies, etc. 2. Introduce ice-breaker and rapport-building activities. Participant’s action: 1. Discuss beneficial effects of a healthy lifestyle. 2. Propose actions to reduce time spent in sedentary behaviours.
		After the session	1. Read the guide’s article about encouraging a healthier lifestyle. 2. Establish personal goals to reduce time spent in sedentary behaviors, based on ideas listed during group session.
1^st^ Individual Session (week 2)	To become aware of the effect of his/her cardiovascular risk factors on his/her health status To establish personal goals for controlling his/her risk factors and plan for the actions needed to succeed in achieving those goals	Before the session	Leader’s action: 1. Email results of baseline tests within 48 h of 1^st^ session. Participant’s action: 1. Read reports of basal tests. 2. Read the guide’s article about metabolic risk factors.
		During the session	1. Discuss reports with the leader. 2. Discuss personal risk factors and establish personal goals for the short (2 weeks), mid (16 weeks), and long term (40 weeks). 3. Select actions to achieve personal goals to reduce metabolic risk factors.
		After the session	1. Place actions needed to achieve risk factor goals in a visible place and start putting them into practice.
2^nd^ Group Session (week 4)	To become aware of the importance of a good workout To establish personal goals to increase physical activity	Before the meeting	1. Read guide’s article about increasing physical activity. 2. Reassess personal level of physical activity according to the reported accelerometer data (guide).
		During the session	Leader’s action: 1. Provide information about ways to achieve a physically active lifestyle. 2. Present specific information about training, hydration, and stretching. 3. Introduce digital resources to help participants be more active. Participant’s action: 1. Discuss beneficial effects of physical exercise in addition to routine physical activity. 2. Brainstorm examples of physical activities and exercise programmes
		After the session	1. Establish physical activity plan to be more active at work and in spare time in the next week. 2. Record physical activity in the smartphone application (app) or on paper.
3^rd^ Group Session (week 7)	To become aware of the possibilities of exercising at home, at work and in spare time using everyday items To establish personal goals to increase physical exercise	Before the meeting	1. Read and watch the video from the guide about tips to be more active at home.
		During the session	Leader’s action: 1. Demonstrate aerobic and strength exercises using stretch bands, dumbbells and improvised exercise material. Participant’s action: 1. Work on aerobic and strength exercises with improvised materials. 2. Share ideas and discuss ways of incorporating physical exercise into daily routines.
		After the session	1. Review the video of the aerobic and strength exercises practiced in class. 2. Establish physical exercise goals and record them in the app or on paper.
4 ^rd^ Group Session (week 10)	To become aware of the importance of good nutrition To establish personal goals to achieve good nutrition	Before the meeting	1. Read the guide’s article about healthy food choices.
		During the session	Leader’s action: 1. Provide nformation about healthy benefits of healthy eating. 2. Present the healthy food pyramid and the Mediterranean diet concept. Participant’s action: 1. Discuss the beneficial effects of healthy nutrition. 2. Brainstorm different ways of making healthy food choices.
		After the session	1. Identify unhealthy food choices made and record them in the app or on paper.
2^nd^ Individual Session (week 12)	To assess the current routine of physical activity and exercise and establish new strategies if necessary To become aware of the effect of good nutrition on his/her health status To establish personal nutritional goals and the actions needed to achieve them	Before the meeting	Leader’s action: 1. Email results of baseline dietary habits questionnaire within 48 h of 2^nd^ session. Participant’s action: 1. Carry out a self-assessment of the establishment of physical activity/exercise goals and the progress made. 2. Read report of baseline dietary habits questionnaire. 3. Read the guide’s article about strategies to overcome nutritional barriers.
		During the session	1. Review progress and resolve problems or setbacks in active lifestyle goal-setting and action planning. 2. Highlight positive changes and define potential ways forward, building on successes. 3. Discuss the dietary habits report with the leader and agree on personal goals. 4. Identify possible barriers to healthy eating and select actions/strategies to overcome them.
		After the session	1. Develop physical activity and exercise plan for the upcoming weeks. 2. Place actions needed to achieve active lifestyle goals in a visible place and start putting them into practice.
6^rd^ Group Session (week 16)	To become aware of barriers to new activity and nutritional behaviors and devise strategies to overcome them To establish new strategies to overcome barriers and maintain a new healthy lifestyle	Before the meeting	1. Read guide’s article about other healthy habits such as sleep and rest, reduce stress, no smoking, etc. 2. Review goals and think about new actions to overcome barriers.
		During the session	Leader’s action: 1. Provide information about maintaining a healthy lifestyle. 2. Resolve doubts and facilitate access to additional resources that may be needed. Participant’s action: 1. Share strategies to overcome barriers to new active lifestyle and dietary habits. 2. Brainstorm ideas to use the physical and social environment to become healthier.
		After the session	1. Review materials, acquired knowledge and strategies. 2. Think about how to apply them in oneself current situation.
3^rd^ Individual Session (weeks 18–19)	To become aware of the effects of oneself new lifestyle on reducing cardiovascular risk factors To establish new weekly personal goals for the next 6 months	Before the meeting	Leader’s action: 1. Email results of post-intervention tests within 48 h of 1^st^ session. Participant’s action: 1. Read the email reports of post-intervention results. 2. Review initial metabolic risk factors results emailed after 1^st^ group session.
		During the session	1. Analyse progress based on 16-week follow-up report after 16 weeks) and resolve problems or setbacks. 2. Highlight positive changes and discuss potential ways forward. 3. Agree on relapse prevention plans (active lifestyle and healthy eating) for the next 6 months.
		After the session	1. Maintain a new healthy lifestyle. 2. Keep a log of physical activity/exercise and eating habits, supported by the BELLUGA’T app. 3. Consult the programme guide, other educational material provided, and physical activity or primary healthcare professionals, as needed.

##### Group counselling sessions

Group sessions involving no more than 10 participants will be scheduled with the aim of providing counselling on a healthy lifestyle, including physical activity, sedentary behaviour and dietary habits. Strategies for behaviour change will also be introduced as a way of empowering participants. Additionally, these sessions will give participants the opportunity to receive peer support.

##### Individual sessions

The aim of the individual sessions will be to focus in greater depth on each participant’s needs. The professional will help the participant to establish realistic daily goals for physical activity and will involve him/her in the decision-making process. The session will be based on the transtheoretical model of behaviour-change [26] and on motivational interviewing [27].

#### Supervised physical exercise

The supervised exercise programme will be offered to both exercise groups (AIT and TCT) and will consist of 16 supervised group training sessions lasting 50 min each and 32 non-supervised individual training sessions (i.e. self-administered by each participant).

The supervised training sessions will be run by experts in physical exercise (sports centre based), who will also deliver the instructions for the self-administered sessions.

##### Structure of the training sessions

All training lessons will comprise five parts: introduction (aims and contents), warm-up, main part (differing between the AIT and TCT groups, see below), cool-down, and stretching and conclusions (instructions for self-administered sessions).

The main part will differ between groups and consists of:

- AIT group: Four series of 4 min of cycling at a heart rate of 80% of the VO_2peak_ registered previously during the stress test, with active pauses of 2 min at 60% of the VO_2peak_ between them (Table 2).

**Table 2:** Weekly schedule for the main part of the AIT program sessions

Week number	Supervised/self-administered lessons (n/n)	Content of the main part of the AIT sessions			
		Work volume (Rep x time)	Work Intensity (% VO_2peak_)	Active rest (time)	Total accumulated time
1	2/1	16 × 1’	80	30”	24’
2	2/1	8 × 2’	80	1’	24’
3	2/1	5 × 3’	80	2’	25’
4	2/1	4 × 4’	80	2’	24’
5	1/2	4 × 4’	80	2’	24’
6	1/2	4 × 4’	80	2’	24’
7	0/3	4 × 4’	80	2’	24’
8	1/2	4 × 4’	80	2’	24’
9	1/2	4 × 4’	80	2’	24’
10	0/3	4 × 4’	80	2’	24’
11	1/2	4 × 4’	80	2’	24’
12	1/2	4 × 4’	80	2’	24’
13	0/3	4 × 4’	80	2’	24’
14	1/2	4 × 4’	80	2’	24’
15	0/3	4 × 4’	80	2’	24’
16	1/2	4 × 4’	80	2’	24’

*AIT* aerobic interval training; n, number, *Rep* repetitions, *VO*_*2peak*_, peak oxygen uptake
- Given that maintaining the anticipated exercise intensity might, at the beginning of the programme, excessively burden participants allocated to this group, the length of the series will be shorter during the initial sessions. Thus, intensity will be established at 80% of the VO_2peak_ but series will last for 1 min during the first week, after which they will be increased by 1 min each successive week until reaching a duration of four minutes. At the same time, active pauses will be shortened so as to accumulate an exercise time of 24 min in that part of the session.
- TCT group: Participants allocated to this group will perform activities such as walking, cycling, team activities and toning exercises, always at an intensity of 60% of their VO_2peak_. The number of training sessions per week and the ratio of supervised to self-administered sessions will be the same as that shown in Table 2 for the AIT group.

##### Programme monitoring

Both training groups will perform the first eight sessions using indoor cycling ergometers, another four lessons using a treadmill and the remainder in outdoor green areas of the city.

Across the sixteen sessions participants will be able to practise and to become familiar with a variety of exercise forms, while keeping to the training goals. The aim is that this experience allows them to feel more empowered with respect to physical activity.

At the beginning of the programme, participants will be given a heart rate monitor (Geonaute, ONRhythm 110, Oxylane, Villeneuve d’Ascq, France) that will be used to monitor the training intensity during supervised and self-administered sessions.

Additionally, a web-based application will be used to monitor and provide feedback for the self-administered training sessions.

#### Educational resources

Educational resources for supporting the counselling programme, such as the participants’ guide [25], have been validated by means of Delphi qualitative techniques [28], patient interview, quantitative questionnaires [29, 30] and the INFELZ software [31].

### Outcome measures

Measurements will be taken before (t0 = 0 months) and after (t1 = 16 weeks) the intervention. As a medium-term follow-up measure, the exercise and control groups will also be assessed 24 weeks later (t2) (Fig. 1). Table 3 shows a detailed outline of the outcome measurements.

**Table 3:** BELLUGAT protocol schedule form and procedures for all interventions

Time point	Enrolment	Baseline assessment	Allocation	+16 weeks assessment	+24 weeks assessment
	t-1	t0		t1	t2
*Preliminary data*:					
Eligibility screen	✔				
Informed consent	✔				
Allocation			✔		
*Anthropometric measurements*					
Body mass		✔		✔	✔
Height		✔		✔	✔
Body mass index		✔		✔	✔
Waist circumference		✔		✔	✔
Hip circumference		✔		✔	✔
*Blood sampling and biochemical measurements*					
Lipid profile (TG, TC, HDLc, LDLc)		✔		✔	✔
Plasma glucose		✔		✔	✔
Plasma glycated haemoglobin		✔		✔	✔
Plasma adiponectin		✔		✔	✔
Plasma Interleucin-6		✔		✔	✔
Plasma metabolome-lipidome		✔		✔	✔
Plasma MicroRNA		✔		✔	✔
*Medical procedures*					
Resting blood pressure		✔		✔	✔
Cardiorespiratory fitness (VO_2_peak)		✔		✔	✔
*Physical activity and sedentary behaviour*					
Physical activity (LPA and MVPA)		✔		✔	✔
Sedentary time		✔		✔	✔
*Self-reported Questionnaires*					
Food frequency questionnaire		✔		✔	✔
Healthy eating index		✔		✔	✔
Empowerment related to health		✔		✔	✔
Physical activity and dietary habits self-efficacy		✔		✔	✔
Health related quality of life (EQ-5D)		✔		✔	✔
Direct costs				✔	
Indirect costs				✔	
Demographic variables		✔			
Enjoyment with the programme				✔	
Reasons for abandoning		✔		✔	✔

*EQ-5D* EQ-5D questionnaire, *HDLc* High Density Lipoprotein cholesterol, *LDLc* Low Density Lipoprotein cholesterol, *LPA* Light Physical Activity, *miRNA* microRNA, *MVPA* Moderate-to-Vigorous Physical Activity, *TC* Total Cholesterol, *TG* Triglycerides, *VO*_*2peak*_ Peak Oxygen uptake

All assessments will be divided into two appointments. In the first appointment, fasting blood samples will be drawn through an antecubital vein, and anthropometric (body mass, height, waist and hip circumferences) and body composition measures (percentage of fat) will be obtained. Also during this appointment, accelerometers will be positioned on the participants’ waist and they will be given instructions on how to complete an activity log during the 7-day period that they will wear the accelerometers.

In the second assessment appointment, participants will be asked to complete the questionnaires on health-related quality of life, self-efficacy, empowerment and dietary habits. Resting (recumbent and sitting) ECG and blood pressure measures will then be taken. Finally, participants will also complete a voluntary maximal graded test on a cycle ergometer.

Accelerometers will be retrieved at the end of the 7-day period during which participants have recorded their free-living movement.

#### Risk factors for metabolic syndrome

##### Anthropometry and visceral adiposity

Body mass (precision: 0.1 kg) and height (precisions (0.1 cm) will be measured with a scale (SECA 711, Hamburg, Germany) and stadiometer (Año-Sayol, Barcelona, Spain), with the participant in underwear and barefoot, following standard procedures [32]. Body mass index (BMI) will be calculated by dividing body mass (kg) by height (m^2^).

Waist circumference (WC) (precision: 0.1 cm) will be measured in triplicate at the end of several consecutive natural breaths, at a level parallel to the floor, at the midpoint between the top of the iliac crest and the lower margin of the last palpable rib in the mid-axillary line. Hip circumference (HC) (0.1 cm) will also be measured in triplicate at a level parallel to the floor, at the largest circumference of the buttocks. Both measures will be made with a stretch‐resistant tape and without constricting [33]. The mean of the three measurements will be used in the waist-to-hip ratio calculation (WtHr).

##### Blood pressure

Blood pressure (BP) will be measured at the level of the brachial artery of the dominant arm using an automated device (Omron M, Omron Healthcare Europe B.V. Hoofddorp, The Netherlands), with participants in a relaxed sitting position. Measurements will be taken in duplicate, 2 min apart. The latter of these will be recorded [34].

##### Plasma biochemistry

Blood samples for the determination of plasma triglycerides (TG), total cholesterol (TC), low-density lipoprotein cholesterol (LDLc), high-density lipoprotein cholesterol (HDLc), glycated haemoglobin (HbA1c) and glucose will be drawn after an overnight fast, with participants in a sitting position. Blood samples will be analysed with automated methods at the laboratory of the Arnau de Vilanova University Hospital (Lleida).

#### Metabolic risk score

A continuous metabolic syndrome risk score (cMSSy) will be calculated, as described by Wijndaele [35]. The score contains the five risk factors considered in the definition of metabolic syndrome [1, 36], namely waist circumference, TG, HDLc, systolic blood pressure (SBP) and plasma glucose.

#### Systemic inflammation and adipose tissue parameters

Fasting blood samples will also be used to determine inflammation parameters (adiponectin and interleukin (IL)-6). Inflammation parameters will be measured by immune assay using high sensitivity *MILLIPLEX®XMap* commercial kits *(*Millipore, Billerica, MA, USA). The visceral adipose tissue index will be calculated following the procedure described by Amato [37]; which relates waist circumference, body mass index, triglycerides and HDLc.

#### Plasma microRNA determination

We will analyse miR-197, miR-23a, miR-509-5p, miR-130a-b, miR-195, miR-27a and miR-320, which are known to be deregulated and associated with features of metabolic syndrome. Targeted microRNA will be determined after its extraction from blood samples, following the instructions provided with the MagMax mirVana RNA isolation kit. In addition, any microRNA will be quantified by a specific TaqMan Advanced miRNA assay.

#### Untargeted and targeted metabolomics and lipidomics

Metabolomics and lipidomics will be performed after the extraction of metabolites and lipids from plasma/serum at the baseline and final stages of the experiments. Metabolites and lipids will be separated by UPLC and detected by QTOF and TQP, depending on the procedure. Species identification will be performed via the PCDL database of Agilent Technologies, which uses retention times in a standardized chromatographic system as an orthogonal searchable parameter to complement accurate mass data (accurate mass retention time approach). In both cases, the SMPDB database [38, 39] will be used for querying metabolites obtained from the HMDB search, using a 0.05 Da M.W. tolerance and the Metaboanalyst platform [40, 41] for the determination of differential features.

#### Cardiorespiratory fitness

Cardiorespiratory fitness will be assessed by means of voluntary maximal graded exercise on a cycle ergometer (Monark 828E, Monark, Sweden).

Before the exercise test, measures will be taken of heart rate and systolic (SBP) and diastolic (DBP) blood pressure, along with a resting electrocardiogram (ECG). Electrocardiographic leads will be positioned according to Mason-Likar placement [42].

These tests will be performed at a constant cadence of 60 rpm. The warm-up stage will be 2 min at 10 W (W). The stepwise graded exercise will then begin, with increases of 20 W every two minutes until volitional fatigue or until participants cannot maintain the pre-established cadence. The recovery stage will be 3 min at the load selected by the participant.

During the graded exercise, oxygen uptake and ventilation will be measured using the Oxycon Mobile metabolic system (Oxycon Mobile, Carefusion, Germany). Gas calibrations will be conducted before each test. Heart rate will be measured using a Polar 610 s chest heart rate monitor (Polar Electro YO, Kempele, Finland) and an electrocardiographic device (Kaunas Load System, Kaunas, Lithuania).

SBP and DBP will be measured using a manual device (Omrom M, Omron Healthcare Europe B.V. Hoofddorp, The Netherlands) at rest, every 2 min during the final 30 s of each stage and at 2, 4 and 6 min of the recovery.

Ratings of perceived exertion (RPE) will be obtained every 2 min using the 10-point Borg scale [43].

Capillary blood samples (200 μL) from the ear lobe will be taken at baseline and at the sixth minute of the post-exercise period for the analysis of plasma changes due to exertion (miRNA determination and untargeted and targeted metabolomics and lipidomics).

Data from the exercise test will be recorded for the screening of abnormal BP and ECG responses, eliciting either VO_2max_ or VO_2peak_, as well as for determining subsequent exercise prescriptions. The criteria for achieving VO_2max_ will be RER >1.1, a plateau in VO_2_ (change of <100 mL · min^−1^ in the final two consecutive stages), and a HR within 10 beats · min^−1^ of the maximal level predicted by age [7]. Those participants who do not meet these criteria will be classified as having reached their VO_2peak_.

The cycle ergometer test is preferred here to the treadmill test because it is easier to take the ECG and BP measurements, and because during the intervention most of the training sessions will be group indoor cycling, and hence the transfer of prescribed heart rate will be more valid. In addition, cycle ergometry is safer than treadmill testing.

#### Physical activity habits and sedentary behaviour

Active and sedentary habits will be assessed objectively by accelerometry using the ActiGraph GT3X+ accelerometer (ActiGraph LLC, Pensacola, FL, USA) over eight consecutive days. Accelerometers will be positioned laterally on each participant’s waist and attached with an elastic belt. Participants will be instructed to wear the accelerometer all day, including during sleep. Accelerometers will be programmed to register the movement in 60-s epochs and data will be downloaded and analysed with ActiLife 6.0 software (ActiGraph, Pensacola, FL, USA). Sleeping hours and 20-min bouts of consecutive zero counts will be excluded from the analysis. Data from accelerometers will be analysed to yield an overall physical activity index (expressed as vector magnitude in mean counts per minute (CPM)) and the percentage of registered time spent in different levels of sedentary behaviour or physical activity (PA). The cut-off points for the categorization of movement intensity will be defined as follows: sedentary behaviour (SB) (<100 CPM); light physical activity (LPA) (100 to 2019 CPM) and moderate-to-vigorous physical activity (MVPA) (>2020 CPM) [44]. Sedentary behaviour will be assessed as the total time engaged in sedentary behaviour and as the daily number and length of sedentary periods. The assessment of physical activity and sedentary behaviour will be complemented with a log of the activities performed during the days on which participants wear the accelerometers.

#### Dietary habits

Data regarding participants’ dietary habits will be obtained using the adult version of the food frequency questionnaire (FFC) [45]. This questionnaire consists of a list of food groups, with participants being required to indicate their intake frequency (daily, weekly or monthly) for each component on the list. This questionnaire has been used previously in longitudinal dietary studies and in studies relating dietary patterns and biological parameters in adults [46].

The *Healthy Eating Index* [47, 48] will be calculated to assess and monitor participants’ dietary status. The Healthy Eating Index (HEI) comprises 10 items, each representing different features of a healthy diet: items 1 to 5 measure the degree of agreement with national dietary guidelines for grains, vegetables, fruit, milk and meat. Items 6 and 7 measure the percentage of total fat and saturated fat in the total energy intake, items 8 and 9 measure cholesterol and sodium intake, and item 10 examines the variety of the respondent’s diet.

#### Empowerment and self-efficacy

Empowerment related to health will be measured with the Spanish version of the Health Empowerment Scale (HES) [49]. This instrument contains 8 items (self-control, self-efficacy, problem solving, psychological coping, stress management, support motivation and decision-making) that are scored on a 5-point Likert scale rating from 5 (strongly agree) to 1 (strongly disagree). Higher scores indicate greater levels of health-related empowerment. The Spanish version of the HES achieved a Cronbach’s alpha (internal consistency) of 0.89, and its content validity is supported by scale and item content validity indexes of 0.98 and 1.0, respectively.

Self-efficacy in relation to physical exercise and dietary habits will be measured with a questionnaire created ad hoc in accordance with the guidelines of Bandura [50]. This instrument comprises 17 items related to physical exercise and 10 items related to dietary habits. Each item is scored on a 100-point scale ranging, in 10-unit intervals, from 0 (cannot do it at all) to 100 (highly certain I can do it). Higher scores indicate greater levels of self-efficacy in relation to exercise and dietary habits.

#### Health-related quality of life

Health-related quality of life (HRQoL) will be determined with the Spanish version of the EuroQoL group EQ-5D questionnaire for adults [51]. The EQ-5D comprises questions related to five dimensions: mobility, self-care, usual activities, pain/discomfort and anxiety/depression. Each dimension has five levels ranging from “no problems” to “extremely important problems”. The EQ-5D also includes a vertical visual analogue scale (EQ VAS) on which respondents self-rate their current perception of health between the endpoints: ‘100 = Best imaginable health state’ and ‘0 = Worst imaginable health state’. The EQ-5D has shown reliability of 0.86 to 0.90.

#### Cost analysis

Direct and indirect costs of setting up and running the interventions will be calculated in order to assess the economic aspect. HRQoL scores will be used to weight survival years and, thus, to generate QALYs (Quality Adjusted Life Years). The cost-effectiveness analysis will be conducted in accordance with the current practice methods for economic assessment [52]. The primary cost-effectiveness outcome will be a reduction in the metabolic risk score.

#### Other measures

##### Demographic and other variables

Variables such as demographic characteristics, personal and family medical history, and medication use will also be registered.

##### Questionnaires used during the intervention phase

During the intervention phase several questionnaires will be used to assess the degree of enjoyment with [53] or the reasons for abandoning the exercise programme.

### Data analysis

#### Timeline of data measurements

Measurements will be taken before (Baseline, t0 = 0 months) and after (t1 = 16 weeks) the intervention. As a medium-term follow-up measure, the two intervention groups and the control group will also be assessed 24 weeks after the end of the programme (t2).

#### Data management

Confidentiality will be ensured by coding participants’ personal data, to which only the researchers and health care professionals will have access.

After randomization, participants will receive an randomization code (unique for each participant and blinded for the statistician). All data will be analysed according to this randomization code. The key to the source data will only be known by the researchers and the research coordinator. The researchers will be familiar with the study procedure, be trained prior to the study and will collect all data for this study.

#### Statistical methods

An initial overview analysis will be carried out to detect any errors and to check the data for normality (Shapiro-Wilks test), this being done for all participants and intervention groups.

Intention-to-treat (ITT) analysis will be performed for all recruited participants who complete the baseline assessment, regardless of whether they subsequently complete the whole protocol. Missing outcome values will be replaced by data registered in the previous assessment using the last observation carried forward (LOCF) technique.

For the baseline comparison of groups we will use the chi-square test (or Fisher’s exact test) for categorical data and the analysis of variance (ANOVA) (or Kruskall-Wallis test) for quantitative data (continuous or ordinal). If significant differences are detected we will use Student’s *t*-test (or Mann–Whitney test) for each pair of groups.

For the analysis of the *intervention* effect two time frameworks will be established: efficacy (at the end of the intervention: t1) and durability (at 24-week follow-up: t2). Change will be calculated as the change from baseline values, both as absolute values and as a percentage adjusted to baseline values. We will adjust repeated measures techniques to study the general behaviour of the outcomes in the follow-up.

Continuous variables will be expressed as mean and standard deviation (SD). Effect size will be estimated as the mean standardized difference between the mean of each group divided by the pooled standard deviation. In accordance with Cohen’s criterion [54], values of 0.2–0.5 represent small differences, 0.5–0.8 moderate differences and >0.8 large differences. Categorical variables will be expressed as counts (n) and percentages (%), unless otherwise specified.

All analyses will be conducted using the Statistical Package for the Social Sciences (SPSS) v17 (SPSS Institute Inc., IL, USA) and the level of significance will be set at *p* < 0.05.

### Risk and burdens

The main expected difficulties are a low adherence rate and the impossibility of blinding the intervention to participants and researchers. However, we will seek to minimize the former by individualizing attention (providing and discussing written feedback reports) during the implementation phase and the latter by blinding researchers to the group to which individuals were allocated.

## Discussion

In its 2008–2013 action plan the WHO [55] states, among others, the following aims: a) the promotion of interventions for the prevention and control of the main modifiable risk factors for non-communicable disease, b) the implementation of actions to help individuals to enhance their self-efficacy, improving literacy and providing tools for self-control, and c) the promotion of research to establish the cost/efficacy relationship of these interventions. We believe that with our study design we focus on these key aspects.

Current evidence indicates that AIT may be a valuable physical exercise strategy in terms of improving cardiac and metabolic risk status, functional capacity and quality of life [12, 18, 20]. AIT has demonstrated consistently better results than TCT when it comes to improving VO_2max_. Given that the VO_2max_ is associated with premature mortality and cardiovascular risk [56] the potential ability of AIT to improve this parameter is, from the health perspective, a promising finding. However, although the efficacy of AIT to induce physiological improvements in a well-controlled research environment is no longer in doubt, questions remain regarding the extent to which its benefits are transferable to a less-controlled clinical environment [21]. A further issue is that the evidence regarding the effectiveness of more traditional programmes for promoting physical activity is also inconsistent [57]. This study aims to address this issue by evaluating the effectiveness of AIT versus TCT (both concomitant with lifestyle counselling) as a primary care intervention for the management of metabolic syndrome risk factors in low-active adults presenting one or more of such factors.

A major strength of our study is its design, which will shed light on the extent to which 1) an AIT programme is a viable tool for implementation in the primary care setting, and 2) whether individuals will remain compliant with the programme in the medium term without supervision. Our study is unique as, unlike other AIT studies, it considers not only the physical exercise aspect but also links this to strategies for behaviour change and empowerment of individuals. According to Biddle [58], structured physical exercise programmes led by competent professionals are more effective at encouraging sedentary individuals to become more active than are poorly structured programmes or programmes that are self-administered at home. However, in order to achieve long-lasting and successful outcomes these programmes need to include psychological goals and strategies (mood, literacy, self-efficacy, social integration) focused on behaviour change. In this regard, our study includes a lifestyle counselling intervention that incorporates strategies for promoting regular physical activity, minimizing sedentary behaviour, improving dietary habits and enhancing participants’ self-efficacy. To our knowledge, this would be the first study in Spain to analyse the effect of AIT in conjunction with strategies to promote behaviour change and empower participants.

Another important feature of our study is that, alongside the assessment of changes in traditional metabolic risk factors after the intervention, we will also examine its effect on systemic inflammatory status [5, 59], cardiorespiratory fitness, self-efficacy for physical activity and dietary habits, empowerment related to health and health-related quality of life, as well as changes in physical activity levels, sedentary behaviour and dietary habits.

As noted by Marrugat et al. [60], the most efficient way of controlling the economic burden of public health and the mortality rates associated with cardiovascular diseases is to reduce their prevalence. One of the strategies for doing so could be the implementation of interventions designed to promote a healthy lifestyle, but in order to establish public health priorities it is first necessary to determine the cost effectiveness of such interventions. Consequently, the present study, in addition to examining the effects on physiological, psychological and behavioural parameters, will also assess the economic impact of the intervention.

The results of this study will provide insight into the immediate and medium-term effects on metabolic risk and lifestyle of the combination of AIT with a multidisciplinary behavioural intervention. If effective, the proposed intervention would provide both researchers and practitioners in this field with a platform on which to develop similar intervention programmes for tackling the repercussions of an unhealthy lifestyle.

## Abbreviations

AIT: Aerobic Interval Training
ANOVA: analysis of the variance
BMI: Body Mass Index
BP: Blood Pressure
cMSSy: Metabolic Syndrome Risk Score
COU: counselling
CPM: Counts Per Minute
DBP: Diastolic Blood Pressure
ECG: Electrocardiogram
EQ VAS: EQ Visual Analogue Scale
EQ-5D: EQ-5D questionnaire
FFC: Food Frequency Questionnaire
HbA1c: Glycated haemoglobin
HC: Hip Circumference
HDLc: High Density Lipoprotein cholesterol
HEI: Healthy Eating Index
HES: Health Empowerment Scale
HIIT: High-Intensity Interval Training
HRQoL: Health Related Quality of Life
IDIAP: Primary Care Research Institute-Jordi Gol
IL-6: interleukin-6
ITT: Intention-To-Treat
LDLc: Low Density Lipoprotein cholesterol
LOCF: Last Observation Carried Forward technique
LPA: Light Physical Activity
miRNA: microRNA
MVPA: Moderate-to-Vigorous Physical Activity
PA: Physical Activity
PCDL: Personal Compound-Database and Library
QALYs: Quality Adjusted Life Years
QTOF: Quadrepole Time-Of-Flight
RER: Respiratory Exchange Ratio
RNA: Ribonucleic Acid
RPE: Ratings of Perceived Exertion
Rpm: Revolutions per minute
SBP: Systolic Blood Pressure
SMPDB: Small Molecule Pathway Database
TC: Total Cholesterol
TCT: Traditional Continuous Training
TG: Triglycerides
TQP: Triple-Quadre-Pole
UPLC: Ultra Performance Liquid Chromatography
VO_2_: Oxygen uptake
VO_2max_: Maximal Oxygen uptake
VO_2peak_: Peak Oxygen uptake
WC: Waist Circumference
WHO: World Health Organisation
WtHr: Waist-to-Hip ratio.
